# Influenza B Virus Ribonucleoprotein Is a Potent Activator of the Antiviral Kinase PKR

**DOI:** 10.1371/journal.ppat.1000473

**Published:** 2009-06-12

**Authors:** Bianca Dauber, Luis Martínez-Sobrido, Jana Schneider, Rong Hai, Zoe Waibler, Ulrich Kalinke, Adolfo García-Sastre, Thorsten Wolff

**Affiliations:** 1 P15, Robert Koch-Institute, Berlin, Germany; 2 School of Medicine and Dentistry, University of Rochester, Rochester, New York, United States of America; 3 Department of Microbiology, Mount Sinai School of Medicine, New York, New York, United States of America; 4 Division of Immunology, Paul Ehrlich Institut, Langen, Germany; 5 Department of Medicine, Division of Infectious Diseases, Mount Sinai School of Medicine, New York, New York, United States of America; 6 Emerging Pathogens Institute, Mount Sinai School of Medicine, New York, New York, United States of America; University of North Carolina, United States of America

## Abstract

Activation of the latent kinase PKR is a potent innate defense reaction of vertebrate cells towards viral infections, which is triggered by recognition of viral double-stranded (ds) RNA and results in a translational shutdown. A major gap in our understanding of PKR's antiviral properties concerns the nature of the kinase activating molecules expressed by influenza and other viruses with a negative strand RNA genome, as these pathogens produce little or no detectable amounts of dsRNA. Here we systematically investigated PKR activation by influenza B virus and its impact on viral pathogenicity. Biochemical analysis revealed that PKR is activated by viral ribonucleoprotein (vRNP) complexes known to contain single-stranded RNA with a 5′-triphosphate group. Cell biological examination of recombinant viruses showed that the nucleo-cytoplasmic transport of vRNP late in infection is a strong trigger for PKR activation. In addition, our analysis provides a mechanistic explanation for the previously observed suppression of PKR activation by the influenza B virus NS1 protein, which we show here to rely on complex formation between PKR and NS1's dsRNA binding domain. The high significance of this interaction for pathogenicity was revealed by the finding that attenuated influenza viruses expressing dsRNA binding-deficient NS1 proteins were rescued for high replication and virulence in PKR-deficient cells and mice, respectively. Collectively, our study provides new insights into an important antiviral defense mechanism of vertebrates and leads us to suggest a new model of PKR activation by cytosolic vRNP complexes, a model that may also be applicable to other negative strand RNA viruses.

## Author Summary

Upon viral infection of vertebrate cells, a vigorous innate defense response is initiated *via* the recognition of viral double-stranded (ds) RNA by the protein kinase PKR, resulting in the cessation of protein synthesis and subsequent blockage of viral propagation. The activation of PKR's potent antiviral response against influenza and other viruses with a negative strand RNA genome has presented a conundrum, however, as previous attempts failed to detect dsRNA in cells infected with these viruses. Here, we identify genomic RNA within the ribonucleoprotein (RNP) of influenza viruses as a non-canonical activator of the latent kinase PKR. Cell biological examinations revealed that the transfer of viral RNP from the nucleus to the cytoplasm provides a strong stimulus for PKR activation. Moreover, we provide insight into mechanisms of pathogenesis by showing PKR and the NS1 protein of influenza B virus forms a complex in infected cells, which inhibits PKR activation. This interaction seems to be crucial for viral pathogenicity, as a strong attenuation of NS1 mutant viruses was largely rescued in PKR-deficient mice and cells. Taken together, these findings suggest a new model for the induction and inhibition of PKR by influenza virus that may also apply to viruses with a similar genome structure.

## Introduction

The presence and replication of viral nucleic acids in vertebrate cells triggers innate immune responses by the activation of antiviral enzymes and induction of type I interferon (IFN) genes [1]. The double-stranded (ds) RNA-dependent protein kinase PKR is a key mediator of this innate immune defense functioning as a signal transducer in a variety of cellular processes [2],[3]. Human PKR is a latent serine/threonine kinase of 551 amino acids with two consecutive N-terminal double-strand (ds) RNA-binding motifs, a linker domain, and a C-terminal kinase domain [4]. PKR is present in non-stimulated cells at basal levels, but its expression is upregulated by type I IFN, which allows a robust response to viral infection [5]. Activation of PKR during infection involves recognition of viral nucleic acids, which induces a structural rearrangement leading to dimerization and autophosphorylation of the kinase at threonine residues 446 and 451 [2]. The best-studied natural target site of activated PKR is serine 51 of the alpha subunit of the eukaryotic translation initiation factor 2 (eIF2α). Its phosphorylation brings about a translational block of cellular and viral mRNAs and hence, a strong impairment of viral replication [2],[3]. In addition, PKR controls transcriptional activation of the nuclear factor-kappa B (NF-κB) pathway and was also shown to mediate apoptosis and to function as a tumour suppressor [3].

Many virus families have evolved gene products targeting PKR, illustrating the high relevance of this kinase in antiviral defense [3],[6]. The inhibitory mechanisms include PKR degradation, sequestration of viral dsRNA by a viral protein, preventing PKR activation through inhibitory viral proteins or viral decoy RNA, and regulating the phosphorylation of eIF2α through a viral pseudosubstrate or recruitment of a cellular phosphatase [3]. Early studies identified dsRNA with a minimum length of 34 base pairs as a prototypical activator of PKR [7] and the sources of these kinase-inducing nucleic acids have been well recognized for several classes of viruses: Complex DNA viruses such as vaccinia virus, adenovirus or herpes simplex virus transcribe open reading frames in opposite orientations leading to formation of duplex RNAs [8]. In reovirus-infected cells, the viral genome consists of dsRNA and also the genomes of many plus-strand RNA viruses contain long stretches of extensively base-paired secondary structure elements [8]. Surprisingly, there is little knowledge about the specific nucleic acids of influenza and other negative-sense RNA viruses that trigger PKR activation, as earlier attempts failed to detect dsRNA in cells infected with such viruses [9],[10]. Progress in this area has been hampered in part by viral suppressors of PKR, which necessitates the application of reverse genetic procedures for such analyses.

The influenza A and B viruses are globally distributed pathogens of the *Orthomyxoviridae* family that cause acute severe respiratory disease and around 40,000 deaths each year in the European Union alone (http://ecdc.europa.eu/Health_topics/influenza/facts.html). These viruses have a segmented genome that consists of eight single-stranded viral RNAs (vRNA) of negative polarity with short complementary sequences at their 5′- and 3′-ends [11]. The vRNAs carry a triphosphate group at their 5′-ends and associate with the viral polymerase complex and the nucleoprotein NP into viral ribonucleoprotein (vRNP). In the nucleus, the viral polymerase synthesizes mRNAs and positive strand cRNAs that serve as templates for new vRNAs. Progeny vRNPs are exported to the cytoplasm in the late phase of infection *via* the CRM1-pathway to facilitate virus assembly and budding at the plasma membrane [11]. It has been suggested that hypothetical dsRNA intermediates produced during virus replication induce PKR [8],[12]. Such a scenario is unlikely, however, as production of viral RNAs with opposite polarities is a nuclear event [11], whereas activation of PKR occurs in the cytoplasm [13]. It was also proposed that influenza virus infection induces PACT, a stress-activated protein activator of PKR [14], but the dependency of PKR induction on PACT expression during viral infection has not been reported yet.

Despite uncertainty regarding the mode of PKR stimulation, it is well established that the influenza A and B virus NS1 proteins (A/NS1 and B/NS1, respectively) function as PKR antagonists since mutant viruses with defects in the NS1 gene, but not wild-type virus, are potent PKR activators [15]–[17]. The NS1 proteins of both virus types are multifunctional proteins consisting of 202–237 and 281 amino acids (aa), respectively. Although their overall sequence identity is below 25%, they carry a similarly structured N-terminal dsRNA binding domain located at positions 1–73 (type A) and 1–93 (type B) [18]. Both NS1 proteins bind to the same RNAs *in vitro* including synthetic dsRNA, U6 RNA, and poly(A)-RNA [19]; however, at present it is unclear whether the NS1 proteins sequester viral dsRNA in the same way as other viral PKR antagonistic proteins, how dsRNA binding is related to PKR inhibition and if the A/NS1 and B/NS1 proteins block PKR activation by the same or different mechanism(s). Interpretation of studies aiming to define the interactions of influenza viruses with PKR and their significance for virus propagation is complex due to the multifaceted nature of NS1 protein function. The NS1 proteins not only inhibit PKR, but also downregulate the signals leading to the activation of type I IFN genes [16], [20]–[26]. Moreover, the A/NS1 protein was shown to inhibit the maturation and export of cellular pre-mRNAs, to enhance translation, to inhibit the 2′-5′- oligo adenylate synthetase (OAS) and to activate the phosphatidylinositol 3-kinase (PI3K) [27]–[35]. In contrast, it is a specific function of the influenza B virus NS1 protein to inhibit the conjugation of the antiviral ISG15 gene product to cellular targets [36],[37]. The B/NS1 protein was recently also shown to modify the nuclear speckle compartment [38]. Thus, a given mutation in the NS1 gene may affect multiple functions, thereby complicating the assignment of a specific host factor as being responsible for a certain phenotype.

In the present report, we propose a novel concept for the induction and control of PKR by influenza viruses that may also apply to other negative strand RNA viruses. Analysis of recombinant viruses showed that activation of PKR is triggered upon the appearance of viral RNP in the cytoplasm late in infection. This reaction was recapitulated *in vitro* by finding that purified viral RNP complexes induce PKR autophosphorylation. Genetic complementation analysis demonstrated that the blockade of PKR by influenza B virus is facilitated by the NS1 protein's dsRNA binding activity, which was also essential for efficient viral replication *in vitro* and *in vivo*. Finally, the antagonistic activity of the NS1 protein was explained by its capacity to form a physical complex with PKR.

## Results

### PKR deficiency rescues attenuated replication and virulence of influenza B mutant viruses with dsRNA-binding deficient NS1 proteins

**Figure 1:**
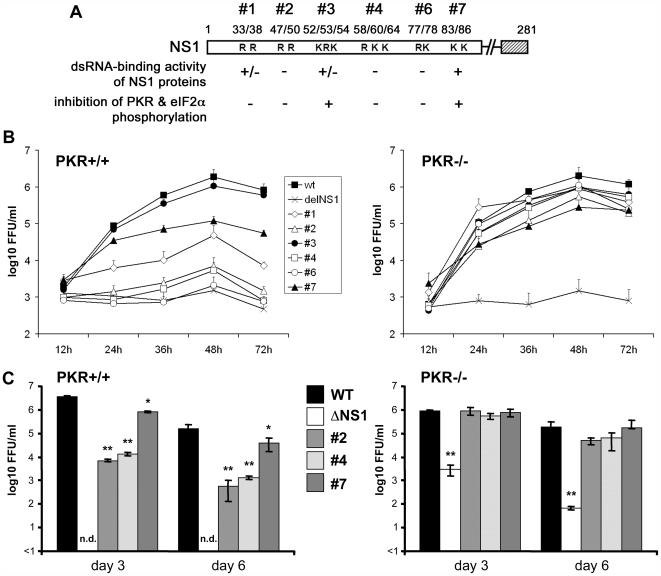
The dsRNA binding activity of the influenza B virus NS1 protein is dispensable for efficient viral replication and pathogenicity in PKR-deficient hosts. (A) The schematic diagram shows basic amino acid residues in the dsRNA-binding domain of the NS1 protein. NS1 proteins with alanine exchange mutations at the indicated positions have been shown to have strong (+), weak (+/−) or no (−) dsRNA binding activity [21]. Recombinant influenza B viruses expressing NS1 proteins with abolished dsRNA-binding and mutant virus #1 did not inhibit PKR activation and eIF2α phosphorylation (−), whereas viruses expressing dsRNA binding NS1 proteins inhibited PKR as WT virus (+) [16]. (B) PKR^+/+^ or PKR^−/−^ MEFs were infected with WT virus, delNS1 virus or NS1 mutant viruses #1, #2, #3, #4, #6 or #7 at an MOI of 0.1. Virus titers were determined at the indicated time points and represent the average of two independent experiments performed as duplicates. Error bars indicate the standard deviation. (C) For infection studies in mice the indicated representative recombinant influenza B/Lee viruses were chosen according to their ability to block PKR activation. Groups of eight-week-old female PKR^−/−^ and wild type C57BL6 mice were anesthetized and infected intranasally with 1×10^5^ ffu of the indicated recombinant influenza B/Lee virus. For viral lung titrations, three mice were sacrificed at day 3 and at day 6 post-infection and virus titers were determined in lung homogenates. Error bars indicate the standard deviation. Statistical analysis indicated significant differences between WT and mutant virus titers. *, p<0.05; **, p<0.01; n.d., not detectable. Other recombinant viruses were not tested in this setting.

Our previous analyses revealed three basic amino acid clusters at positions 47/50, 58/60/64 and 77/78 in the N-terminal region of the influenza B virus NS1 protein to be essential for dsRNA binding as well as for inhibition of PKR and eIF2α phosphorylation [16],[21] (Fig. 1A). Hence, *loss-of-function* viruses expressing NS1 proteins with alanine replacements at those essential positions (mutants #2, #4, #6) or at amino acids 33/38 (mutant #1, dsRNA binding reduced) were attenuated for replication in IFN-competent hosts by several orders of magnitude [16]. In contrast, viruses expressing NS1 proteins with alanine replacements at positions 52/53/54 or 83/86 (mutants #3 and #7), which retained dsRNA binding, inhibited PKR activation and replicated to high titers [16].

To address the question of whether PKR or another antiviral factor was mainly responsible for the attenuation of viruses with dsRNA binding-defective NS1 protein, we conducted a growth curve analysis in embryonic fibroblasts from *PKR^+/+^* and *PKR^−/−^* mice (Fig. 1B). The WT and control mutant virus #3 replicated equally well in *PKR^+/+^* and *PKR^−/−^* cells reaching titers of 1–2×10^6^ FFU/ml. Replication of mutant virus #7 was slightly lower, but this characteristic was independent of the PKR status. In contrast, replication of mutant viruses #2, #4 and #6 expressing dsRNA-binding defective NS1 proteins was reduced in normal mouse embryonic fibroblasts (MEF) by about three orders of magnitude (1.5–7.1×10^3^ FFU/ml) in comparison to WT. The mutant virus #1 replicated to a slightly higher titer (4.9×10^4^ FFU/ml). Significantly, the absence of PKR strongly boosted replication of all four mutant viruses (#1, #2, #4 and #6) to titers between 5.4×10^5^ and 1.1×10^6^ FFU/ml. Interestingly, minimal replication of the isogenic virus with a complete deletion of the NS1 gene (delNS1 virus) was observed both in the absence and presence of PKR, pointing to a vital NS1 function beyond the inhibition of PKR.

To evaluate NS1 dsRNA binding activity *in vivo*, we compared the growth of WT and selected mutant viruses in the lungs of wild-type and *PKR^−/−^* mice [39] three and six days after intranasal infection with 1×10^5^ FFU (Fig. 1C). This dose was the highest applicable amount possible, due to the low growth of some of the mutant viruses. Wild-type mice had strongly reduced lung titers (by about two orders of magnitude) of the mutants expressing dsRNA-binding deficient NS1 proteins (#2 and #4), in comparison to WT virus and control mutant #7; even so, all three mutant viruses replicated in *PKR^−/−^* mice to a similar extent as the WT. The delNS1 virus was not detected at all in normal mice. However, visible delNS1 virus titers were determined in the lungs of *PKR^−/−^* animals, which were, however, considerably reduced compared to WT virus. A similar strong attenuation of replication in PKR^−/−^ mice was recently reported for an influenza B/Yamagata/88 mutant virus expressing a severely truncated NS1 protein of 18 amino acids [40]. The differences in delNS1 virus growth *in vitro* and *in vivo* might be due to a higher permissiveness of the murine respiratory tract in the absence of PKR compared to fibroblasts, or the slightly different genetic backgrounds of the PKR-deficient animals and cells studied. All WT mice survived the challenge with each virus, but there were marked differences in the body weights of infected animals (Fig. S1A). WT and the control mutant virus #7 caused a transient weight loss of up to 15%, whereas there was little or no weight reduction in mice infected with the mutants #2, #4 or delNS1 virus. Interestingly, *PKR^−/−^* mice rapidly lost weight and succumbed to infection between day 7 and 9 after challenge with the WT and all four viruses expressing mutant NS1 proteins, but there was little weight loss in PKR-deficient mice after delNS1 infection (Fig. S1B). These findings demonstrate that PKR can strongly inhibit replication of influenza B virus; however, PKR can be impeded by the dsRNA binding function of the viral NS1 protein.

### DsRNA-binding NS1 proteins form an RNase-sensitive complex with PKR in infected cells

**Figure 2:**
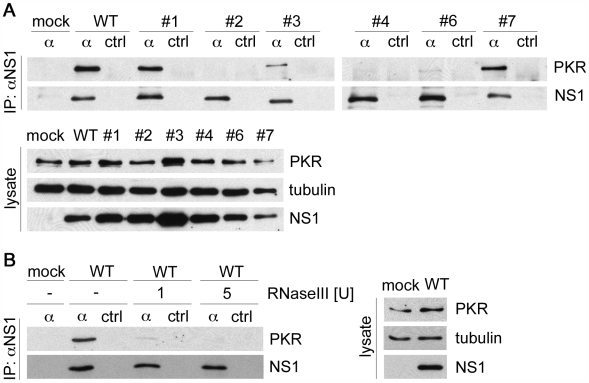
The dsRNA-binding activity of the NS1 protein mediates interaction with PKR. (A) Human A549 lung epithelial cells were mock treated or infected with WT virus or the NS1 mutant viruses #1, #2, #3, #4, #6 or #7 at an MOI of 1. Lysates were subjected to immunoprecipitation (IP) with NS1 antiserum (α) or pre-serum (ctrl). The precipitated complexes were analyzed by immunoblotting with antibodies specific for PKR (upper panel) and NS1 (lower panel). Whole cell lysates were also analyzed with antibodies specific for PKR, tubulin and NS1 as indicated. (B) A549 cells were mock treated or infected with WT virus at an MOI of 1. Lysates were prepared that were either mock treated (−) or treated with the indicated amounts of RNase III for 10 minutes at 33°C prior to immunoprecipitation with NS1 antiserum (α) or pre-serum (ctrl). The precipitates were analyzed as described in panel 2A (panel to the left). Aliquots of the lysates were also analyzed directly by immunoblotting for PKR, tubulin and NS1 as indicated (panels to the right).

To test the hypothesis that NS1 forms a complex with PKR involving dsRNA, we first immunoprecipitated lysates of WT and mutant virus-infected human A549 cells with anti-NS1 serum (Fig. 2A). Immunoblot analysis showed that PKR was specifically coprecipitated with dsRNA binding NS1 proteins expressed by the WT and mutant viruses #1, #3 and #7. In contrast, very little or no PKR was detected in precipitates containing the dsRNA binding-deficient NS1 proteins of the mutant viruses #2, #4 and #6, that fail to prevent activation of PKR [16],[21]. Further analysis showed that pre-treatment of lysate with dsRNA-specific RNase III eliminated the detection of WT NS1-PKR complexes in a dose-dependent manner (Fig. 2B). These results suggest that dsRNA-binding of the NS1 protein is a prerequisite for complex formation with PKR.

### Wild-type but not dsRNA binding-deficient NS1 protein co-sediments with PKR and viral RNP

**Figure 3:**
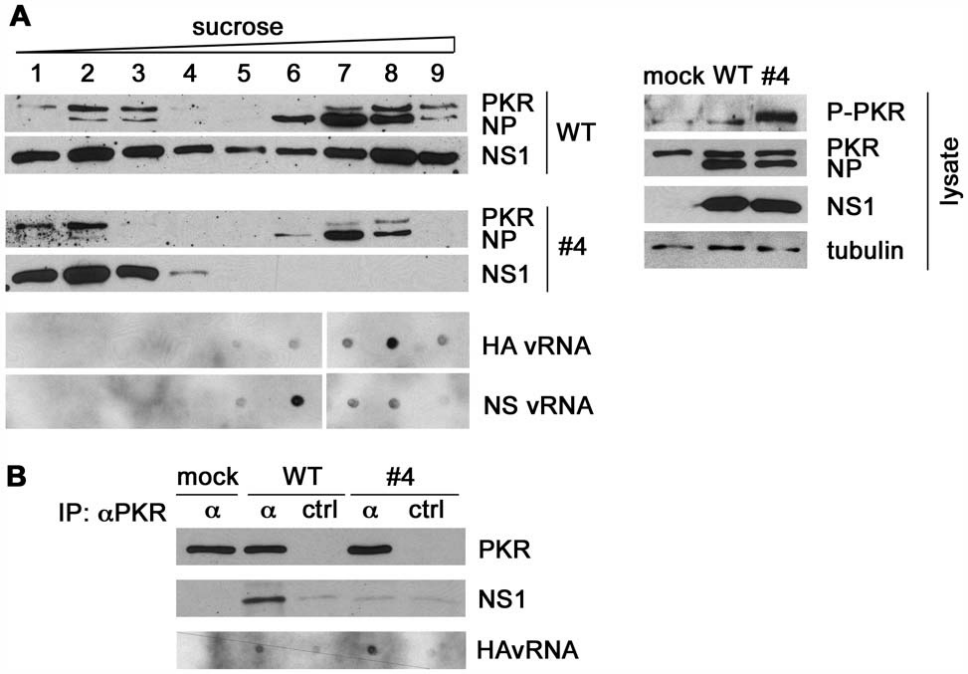
NS1 WT but not dsRNA-binding deficient NS1 protein co-sediments with PKR and vRNP upon density gradient centrifugation. (A) A549 cells were infected with WT virus or mutant virus #4 at an MOI of 1. Cells were lyzed 12 hours p.i. and subjected to centrifugation through a continuous 5 to 50% sucrose gradient. 16 fractions were taken from top to bottom. Fractions 1 to 9 were analyzed by immunoblotting with antibodies specific for PKR and the viral NP and NS1 proteins. Also, RNA was extracted from gradient fractions 1 to 9 and was subjected to dot blot hybridization with probes specific for HA vRNA and NS vRNA, respectively (panels “HA and NS vRNA”). Whole cell lysates were analyzed by immunoblotting with antibodies specific for phospho-PKR, total PKR, viral NP, viral NS1 and tubulin as indicated (right panel,“lysate”). (B) A549 cells were mock treated or infected with WT virus or virus mutant #4 as described in panel A. Lysates were prepared and subjected to immunoprecipitation with anti-PKR (α) or control antibody (ctrl). The precipitated complexes were analyzed by immunoblotting for PKR and NS1 proteins. RNA was isolated from an identical set of PKR immunoprecipitates of cells infected with the mutant virus and subjected to dot blot analysis with an RNA-probe specific for HA vRNA.

To characterize the intracellular complexes containing PKR and NS1 protein, we fractionated lysates of A549 cells infected with the WT or NS1 mutant #4 virus by sucrose density gradient centrifugation (Fig. 3A). Immunoblot analysis of fractions 1 to 9 (top to bottom) showed that the NS1 WT, but not the dsRNA binding-deficient mutant protein co-sedimented with PKR and NP in higher order complexes in fractions 7 to 9 (Fig. 3A). Dot-blot analysis detected genomic viral RNA of the HA and NS segments in the same fractions, indicating the presence of vRNPs (Fig. 3A, panels “HA and NS vRNA”). As expected, control blots showed in lysates of mutant virus-infected cells the presence of phospho-PKR, whereas little activation occurred with WT virus (Fig. 3B, right panel, “lysate”). Since PKR co-sedimented with vRNP the question arose whether these factors interact and if this interaction could be involved in PKR activation. Therefore, we immunoprecipitated lysates of WT and mutant virus-infected cells with PKR-specific antibody and examined the precipitates by immunoblotting and RNA hybridization (Fig. 3B). Efficient co-precipitation of the wild-type NS1, but not the mutant protein was observed (Fig. 3B). We also detected the negative-stranded viral RNA, a major component of the viral RNP, in the precipitates without difficulty. Hence, these results indicate that PKR associates with viral RNP and, depending on intact dsRNA binding, also with the NS1 protein.

### Activation of PKR correlates with cytoplasmic accumulation of vRNPs

**Figure 4:**
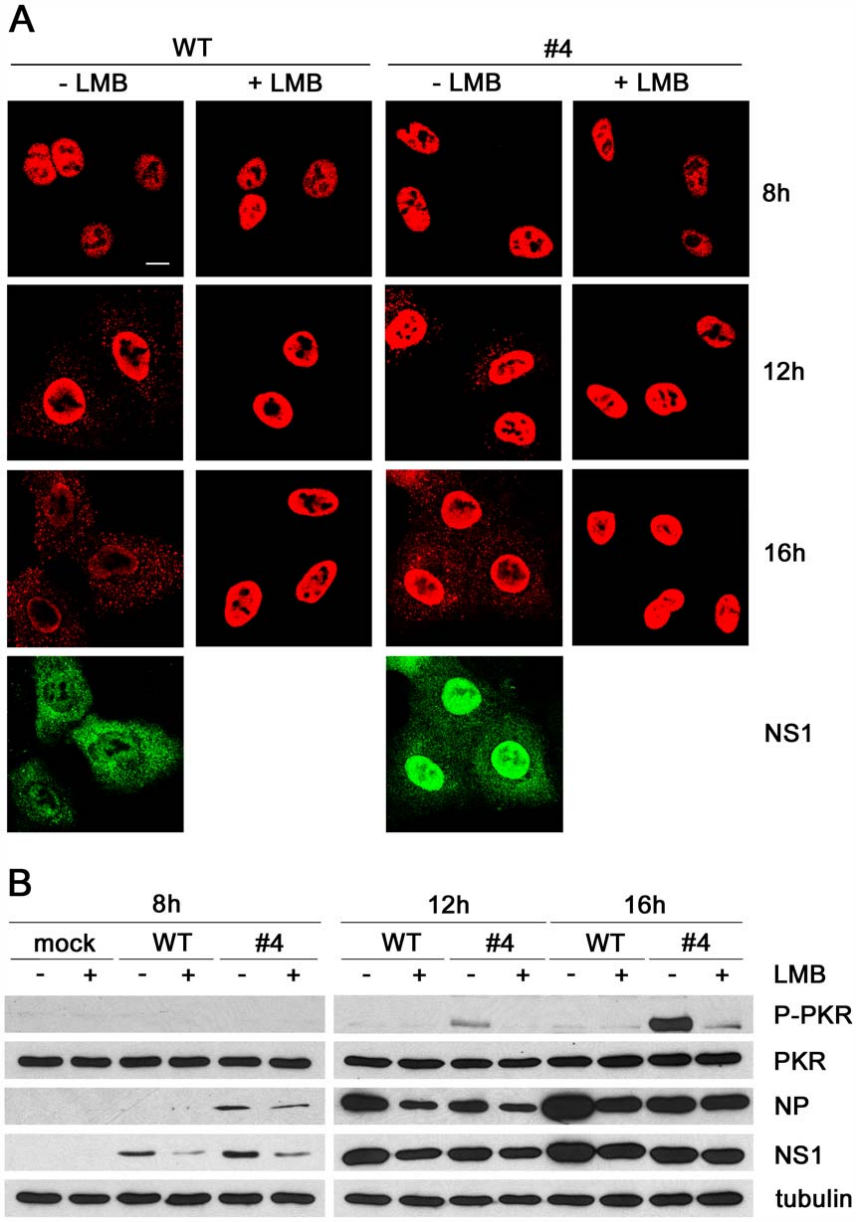
The nuclear egress of vRNPs late in infection leads to PKR activation. (A) A549 cells grown on glass cover slips were infected with WT or mutant virus #4 at an MOI of 1. Cells were mock treated or complemented with LMB starting at 3 hours p.i.. At 8, 12 and 16 hours p.i., cells were fixed and stained for NP (shown in red color), and also for the NS1 protein at the 16 h time-point (shown in green color). Microscopic sample analysis was conducted by confocal laser scanning microscopy. Scale bar, 10 µm. (B) A549 cells grown in culture dishes were infected with WT virus or mutant virus #4 at an MOI of 1. Cells were mock treated or complemented with LMB starting 3 hours p.i. and lyzed 8, 12 and 16 hours p.i.. Whole cell lysates were analyzed by immunoblotting with antibodies specific for phospho-PKR, total PKR, NP, NS1 and tubulin as indicated.

Next, we compared the activation of PKR after infection of cells with the NS1 *loss-of-function* mutant #4 and WT virus in relation to the intracellular localization of vRNP complexes (Fig. 4). Confocal microscopy detected vRNPs as stained by NP-specific antibody in the nucleus at 8 hours post infection (p.i.), but increasing cytosolic signals appeared at 12 and 16 hours p.i. for both viruses (Fig. 4A). At the later time-points, the NS1 proteins were detected in the nuclear and the cytoplasmic compartments, although we noted a slightly stronger nuclear signal for the mutant protein (Fig. 4A). Starting at 12 hours p.i., the cytosolic appearance of vRNP was paralleled by detection of activated PKR in cells infected with the mutant, but not the WT virus (Fig. 4B). A similar picture was observed in cells infected with the delNS1 mutant virus (data not shown). Intriguingly, PKR activation by the mutant #4 virus was strongly reduced in the presence of leptomycin B (LMB), a fungal drug that specifically inhibits the CRM1 pathway and thus the nuclear export of vRNP [41] (Fig. 4A). Immunoblotting for NP, NS1 and tubulin showed comparable viral protein synthesis in the presence of LMB (Fig. 4B) and cell fraction analysis confirmed that LMB strongly reduced cytosolic NP levels under these condition (Fig. S3). These findings suggest that activation of PKR is associated with the cytosolic appearance of vRNPs late in infection.

### Purified viral RNPs trigger PKR kinase activity *in vitro*

**Figure 5:**
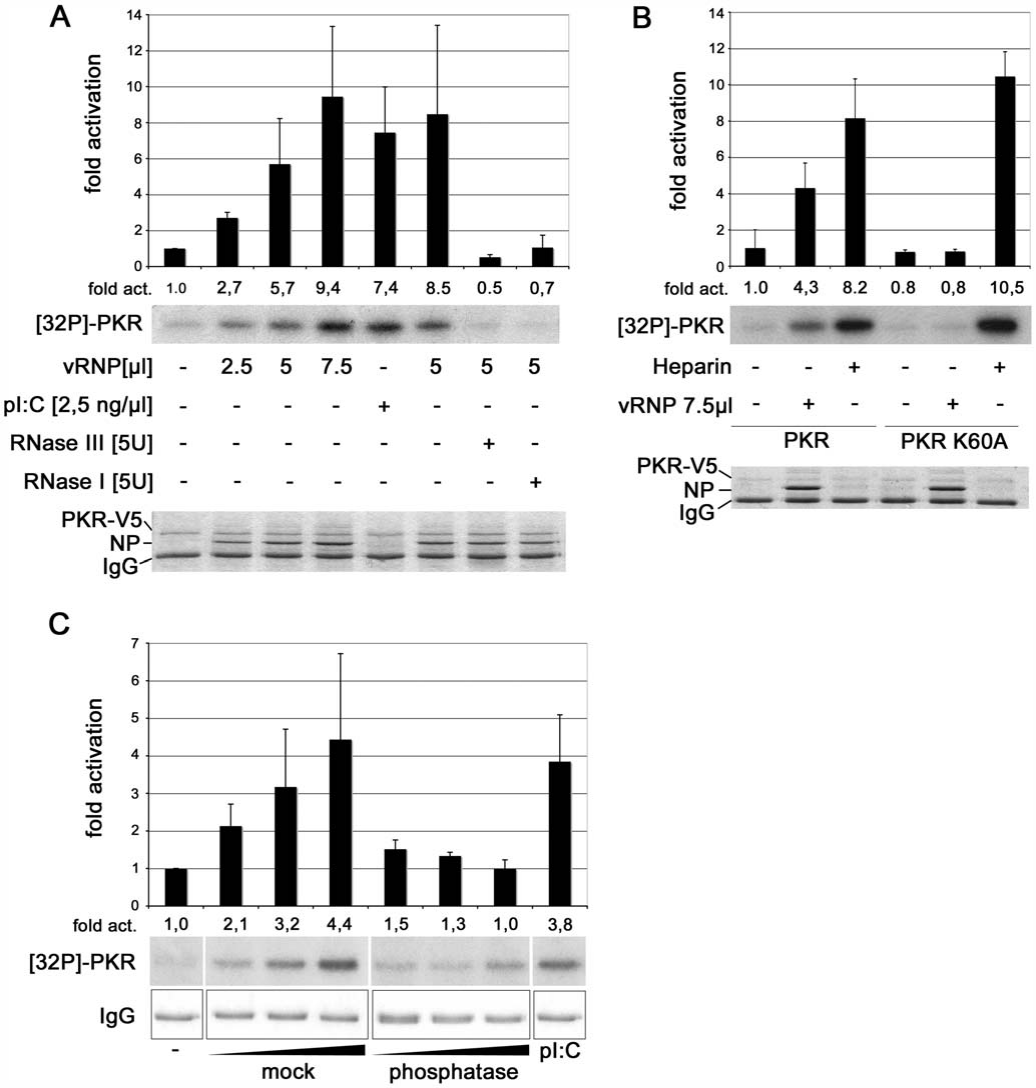
Purified viral RNP activates PKR autophosphorylation *in vitro*. (A) V5-tagged PKR was expressed in 293T cells and precipitated from lysate by anti-V5 antibody. Kinase activation was assayed on precipitated PKR in the presence of 10 µCi [γ-^32^P]ATP for 30 min at 30°C. PKR was activated as indicated by 2.5, 5 or 7.5 µl vRNP, or 2.5 ng/µl pI∶C. In further reactions, 5 µl vRNP were pre-treated with 5 U RNase III or 5 U RNase I, respectively. Kinase reactions were analyzed by SDS gel electrophoresis and autoradiography (upper panel). Autophosphorylation of PKR was evaluated using the AIDA software and is given as fold activation compared to mock treated PKR. The values represent the average of three independent experiments. Error bars indicate the standard deviation. After exposure the SDS gel was stained with Coomassie blue to visualize precipitated V5-PKR, NP and the IgG heavy chain (lower panel). (B) V5-tagged PKR and V5-tagged mutant PKR-K60A that does not bind dsRNA were expressed in 293T cells, precipitated and stimulated in the autophosphorylation assay by vRNP or treatment with heparin as indicated. Quantification was done as described in panel A. (C) V5-tagged PKR was expressed in 293T cells, precipitated and subjected to the kinase activation assay as described above. PKR was incubated as indicated without or with 0.3, 1.0 or 3.0 µM of a transcribed model vRNA of 53 nucleotides, which was mock-treated or dephosphorylated by phosphatase, respectively, as indicated.

To test the hypothesis that influenza virus RNP activates PKR directly, we purified vRNPs from influenza B/Lee virions (Fig. S2) and tested them for stimulation of PKR autophosphorylation. V5 epitope-tagged PKR was expressed in 293T cells and precipitated from cell lysate with tag-specific antibody. Activation of the precipitated kinase in the presence of vRNP was assessed by incubation with [γ-^32^P]-ATP followed by analysis on SDS gels and autoradiography. Fig. 5A shows an exposure of autophosphorylated PKR (lanes “32P-PKR”) and the same region of the gel after staining with Coomassie Blue (Lanes “PKR-V5”, “NP” and “IgG”). Interestingly, the purified vRNP stimulated PKR autophosphorylation in a dose-dependent manner similar to the synthetic dsRNA polyriboinosinic: polyribocytidylic acid (pI∶C) (Fig. 5A). Pre-treatment of vRNP with either dsRNA-specific RNase III or ssRNA-specific RNase I prior to the kinase reaction abolished PKR activation, which argues against the presence of a protein activator of PKR in the reaction (Fig. 5A). Significantly, influenza virus RNP hardly activated the dsRNA binding-deficient PKR-K60A mutant, further confirming the dsRNA-dependency of vRNP-mediated kinase stimulation (Fig. 5B). Control reactions showed that the PKR-K60A mutant enzyme was activated by heparin, a common trigger for PKR autophosphorylation [3], excluding the possibility that the mutation affected general kinase activity. We note that influenza virions package only RNPs containing negative-stranded genomic RNA and not complementary nucleic acids [42], which could induce double-stranded hybrids. Moreover, the kinase assay conditions used prevented the viral polymerase from transcribing complementary RNA, which could hypothetically induce duplex RNA.

It is difficult to determine whether the removal of the 5′-triphosphate group on the vRNA within the native RNP reduces PKR stimulation [43]. Phosphatase not only hydrolyzes the 5′-triphosphate, but also removes the phosphate groups on activated PKR and the donor nucleotide, and a subsequent elimination of the phosphatase by phenol extraction would obstruct RNP structure. However, we showed that phosphatase treatment strongly reduced activation of PKR by synthetic influenza virus model vRNA containing the terminal 5′- and 3′- ends, indicating a contribution of the 5′-triphosphate (Fig. 5C). In conclusion, these experiments suggest that influenza virus RNP, most likely *via* structured regions within the genomic RNA and its unmodified 5′-end, activates PKR.

## Discussion

The present study showed that PKR is a major factor restricting the growth of influenza B virus *in vitro* and *in vivo* and revealed mechanisms for its activation and subversion. We previously reported that mutational inactivation of the dsRNA binding domain of the B/NS1 protein attenuated viral replication and ablated the control of PKR and eIF2α phosphorylation, but only slightly affected IFN suppression [16]. Our new analysis firmly establishes that the key activity of the B/NS1 dsRNA binding domain is to silence PKR and excludes the possibility that another antiviral factor(s) is chiefly responsible for the observed growth defects of the mutants: The absence of PKR restored a virulent phenotype of the mutant viruses expressing dsRNA binding-deficient NS1 protein in mice and viral titers were elevated to the range of wild-type virus in the lungs of *PKR^−/−^* mice and fibroblasts. Interestingly, this is slightly different to a reported main function of the dsRNA binding domain of the influenza A virus NS1 protein in targeting the antiviral 2′-5′-OAS/RNase L system [32]. Our study revealed a further distinction between type A and B influenza viruses since the B/delNS1 virus behaved benignly in both *PKR^+/+^* and *PKR^−/−^* mice, whereas a comparable A/delNS1 virus regained virulence in PKR *null* mice [15]. This finding points to the existence of an additional important function of the B/NS1 protein during viral replication, which is possibly related to its recently reported activity in modifying the nuclear speckle compartment of the host cell [38].

The interactions of PKR with its RNA effectors and viral inhibitors are complex and incompletely understood. Our analysis of defined *loss-of-inhibition* mutant viruses offers new insights into the conundrum of how dsRNA-dependent PKR is activated during infection by influenza virus that does not generate long duplex RNA [10]. Based on several lines of evidence, we put forward the hypothesis that influenza virus RNP functions as a non-canonical activator of PKR in the cytosol: First, PKR autophosphorylation in infected cells occurred concomitantly with the cytosolic appearance of vRNP, when a functional NS1 protein was absent. Second, PKR activation was largely abolished when the nucleo-cytoplasmic export of vRNP was blocked by LMB treatment. Third, biochemical analysis demonstrated that purified vRNP activates PKR in an *in vitro* kinase assay directly. The RNA-dependency of this stimulation was shown by its sensitivity to pre-treatment with single- and double-strand-specific RNases and the failure of the vRNP to activate a dsRNA binding-defective PKR. We favour the explanation that the vRNA promoter structure formed by the partially complementary 5′- and 3′-ends within vRNP is an important determinant for PKR activation. Synthetic A-form RNA consisting of a short stem region flanked by single-stranded nucleotides was previously shown to efficiently stimulate PKR *in vitro* [44]. There is now ample evidence from structural, biochemical and functional studies that the 14–16 nucleotides of the vRNA termini engage in base-pairing interactions and exist in form of a panhandle and/or a related corkscrew structure [45]–[47] (summarized in [11]). Intriguingly, the 5′-ends of influenza virus RNAs carry a triphosphate structure [11] and this chemical group was recently reported to support activation of PKR by stem-loop RNA [43]. In fact, the present study is the first characterization of a natural viral RNA/RNP with a 5′-triphosphate group that triggers PKR, as the studies mentioned above tested exclusively synthetic model RNAs. Our mechanistic model suggests PKR can contact RNA residues within the vRNP despite their association with the viral NP and polymerase proteins.

A second novel aspect of our study centres on the mode by which the influenza B virus NS1 protein inhibits PKR. Previously, we showed that dsRNA binding mutations within NS1 prevented PKR inhibition, but did not eliminate the suppression of IFN induction that is triggered through the RNA helicase RIG-I [9],[16],[48]. Our new study explains the former observation by showing that the NS1 protein entangles PKR into an immunoprecipitable complex in infected cells. These PKR-NS1 complexes were sensitive to treatment with dsRNA-specific RNase and the interaction required a functional NS1 dsRNA binding domain. The most likely interpretation of these findings is that RNA to which both proteins bind facilitates the interaction of PKR and NS1. Interestingly, the association with PKR appears to be necessary but not sufficient to preclude its activation. In fact, all NS1 mutants that were not associated with PKR failed to block kinase activation (this study; [9],[16],[48]), but the NS1 mutant #1 did not prevent PKR activation even though it interacted with the enzyme. Clearly, more work is required to determine the whole catalogue of events leading to the blockade of PKR by influenza B virus. Slightly different modes of PKR association, which did not require dsRNA binding in GST pull-down assays were suggested for the A/NS1 protein [49],[50]. We have therefore initiated comparative studies to determine whether the two divergent NS1 proteins employ different or similar ways to block PKR.

At present, we can only speculate about the nature of the RNA component in NS1-PKR complexes in influenza B virus-infected cells. The presented data argue for a scenario in which cytoplasmic vRNP complexes are recognized by PKR and provide the major stimulus for its activation. We detected both vRNA and the NS1 protein in PKR immunoprecipitates from infected cell lysate raising the possibility that NS1 prohibits activation of PKR by cytosolic vRNP through the formation of a hetero-trimeric complex. However, we cannot rule out the option that binding of NS1 to PKR in infected cells is mediated by a yet undetermined viral or host-derived nucleic acid; for that reason more work will be required to elucidate the specific components involved.

**Figure 6:**
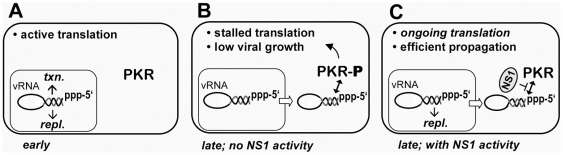
Model for the induction and inhibition of antiviral PKR by influenza virus. The three panels show the localization of the genomic vRNA/RNP complexes with structured 5′- and 3′-ends together with the phosphorylation status of PKR in early (panel A) and late phases (panels B and C) of virus infection. Viral RNP is located in the nucleus early in infection and is not accessible for cytosolic PKR (panel A). Upon nuclear export at later stages, vRNP complexes stimulate PKR activation in the absence of functional NS1 protein, leading to an inhibition of viral proteins synthesis and replication (panel B). However, the presence of functional viral NS1 protein suppresses this antiviral reaction thereby supporting high-level viral propagation (panel C).

The influenza B virus non-structural NS1 protein has apparently evolved in a way that suits the specific needs of the pathogen to inhibit PKR (Fig. 6). During the first hours of infection viral RNPs are confined to the nucleus and not accessible for the cytosolic sensor (Fig. 6A). At later stages, the vRNPs are exported to the cytoplasm where they assemble into progeny virions at the plasma membrane. Cytoplasmic vRNPs act as PKR activators in the absence of functional NS1 protein, thus reducing protein synthesis and virus growth (Fig. 6B). However, in the wild-type situation NS1 protein is expressed and prevents activation of PKR by vRNPs, thereby facilitating full-level synthesis of structural viral proteins and efficient viral propagation (Fig. 6C). This scenario is supported by the dynamic intracellular trafficking of the B/NS1 protein showing that it migrates to nuclear speckles early in infection, but relocalizes to the cytoplasm at later time-points [38]. The model may also explain phenotypes of mutant influenza viruses with lesions in the NS1 protein that activate PKR and show a selective reduction in the synthesis of late viral gene products [16],[17],[51].

Finally, we suggest that activation of PKR by other negative strand RNA viruses that generate little or no detectable long duplex RNA may occur in a similar way. The genomes of many of these viruses enter the cytoplasm as part of an RNP structure and carry a 5′-triphosphate group. In fact, the conserved ends of the genomic RNAs of several members of the *Bunyaviridae* also form a structured panhandle [52] and the level of phosphorylated eIF2α increased during infection with the prototypic Bunyamwera virus [53]. Further observations suggest that also non-segmented negative-strand RNA viruses may induce PKR by a ribonucleoprotein structure; for instance, the Ebola virus VP35 protein counteracts stimulation of PKR, indicating indirectly that members of the *Filoviridae* family activate and prevent induction of the kinase [54]. In addition, replication and virulence of vesicular stomatitis virus, a member of the *Rhabdoviridae*, is strongly enhanced in PKR-deficient mice [55]. Exploring the methods used by viral pathogens to counteract PKR not only promises an improved understanding of the innate immune defense against viral infections, but may also foster the development of novel therapeutic substances. Our study suggests that it should be possible to shift the course of influenza virus infection from efficient pathogen replication towards its elimination *via* the body's innate defense if the NS1-mediated blockade of PKR could be suspended. The recent description of substances capable to modulate NS1's activity in IFN suppression supports the concept that this viral protein is indeed amenable to control by therapeutic substances [56].

## Materials and Methods

### Cells and viruses

293T and A549 cells were grown in Dulbecco's modified Eagle medium (DMEM) supplemented with 10% fetal calf serum (FCS), 2 mM l-glutamine, and antibiotics. Madin-Darby canine kidney (MDCK) type II cells were grown in minimal essential medium (MEM) with the same additives. MEFs were prepared from C57BL/6 mice (*PKR* ^+/+^) or mice devoid of functional PKR (*PKR* ^−/−^) [39] and cultured in DMEM supplemented with 10% FCS, 2 mM l-glutamine and 50 µM β-mercaptoethanol. All cells were maintained at 37°C and 5% CO_2_. The recombinant influenza B/Lee WT and mutant viruses ΔNS1, NS1 33/38 (#1), NS1 47/50 (#2), NS1 52/53/54 (#3), NS1 58/60/64 (#4), NS1 77/78 (#6), and NS1 83/86 (#7) have been described elsewhere [16],[20]. To analyze viral replication, monolayer cultures of *PKR* ^+/+^ or *PKR* ^−/−^ MEFs were infected at a multiplicity of infection (MOI) of 0.1 and incubated at 33°C in DMEM containing 0.2% bovine albumin and 0.5 µg/ml trypsin. Virus titers were determined by indirect immunofluorescence staining and were expressed as fluorescence-forming units (ffu)/ml as described before [20].

### Infections of mice

Eight-week-old female wild type C57BL6 *PKR^+/+^* and *PKR^−/−^* mice with the mixed 129/Sv(ev)×C57BL/6J background [39] were anesthetized and infected intranasally with 50 µl of phosphate-buffered saline (PBS) containing 1×10^5^ ffu of the indicated recombinant influenza B/Lee viruses. For viral lung titrations, three mice were sacrificed at day 3 and at day 6 post-infection. Mice lungs were homogenized in 1 ml PBS, and titrated by immunofluorescence using a monoclonal antibody against influenza B virus nucleoprotein (Abcam; ab20711-100). Comparison of viral lung titers in WT and mutant virus-infected mice at each time-point was done using the Student's t test for pairwise comparisons. For monitoring of viral disease, 8 animals were weighed daily for two weeks and euthanized when observed in extremis. Mice were bred and maintained at the Mount Sinai School of Medicine in accordance with Federal and university guidelines.

### Plasmids

pcDNA3.1-PKR-V5/His was constructed by integration of the human PKR cDNA into the vector pcDNA3.1-V5/His (Invitrogen). The derived pcDNA3.1-PKR-K60A-V5/His plasmid was constructed with the QuikChange mutagenesis kit (Stratagene). The integrity of the constructs was confirmed by DNA cycle sequencing.

### Immunoprecipitation and immunoblot analysis

2.5×10^6^ A549 cells were mock-treated or infected with WT, ΔNS1 or NS1 mutant virus at an MOI of 1. Cell extracts were prepared 8 hours p.i. in IP lysis buffer (1% Igepal-CA 630, 150 mM NaCl, 20 mM Tris·HCl, pH 7.5, 1 mM EDTA, 10 mM Na-β-glycerophosphate, 2 mM Na_3_VO_4_, 1 mM Pefabloc). When indicated, lysates were pre-incubated with RNase III (Ambion) for 10 min at 33°C before incubation with B/NS1-specific rabbit antiserum [16]. Immune complexes were collected on protein-G-agarose beads (Roche), washed and the precipitated proteins were dissolved in SDS sample buffer. For PKR immunoprecipitation cells were lyzed in kinase binding buffer (20 mM Hepes pH 7.5, 300 mM NaCl, 5 mM Mg(OAc)_2_, 10% glycerol, 25 mM Na-β-glycerophosphat, 2 mM Na_3_VO_4_, 1 mM Pefabloc) containing 0.5% Igepal-CA 630. PKR was precipitated with rabbit anti-PKR antibody (Epitomics) and immunocomplexes were collected as described above. The precipitated proteins were analyzed as indicated by SDS gel electrophoresis and immunoblotting using the primary B/NS1-specific rabbit antiserum, mouse anti-PKR antibody 71/10 (Ribogene) or rabbit anti-PKR antibody (Epitomics), mouse anti-NP antibody (AbD Serotec), rabbit anti-phospho-PKR (Thr446) antibody (Cell Signaling), mouse anti-tubulin antibody (Sigma) and suitable secondary horseradish peroxidase (HRP)-conjugated IgG together with an enhanced chemiluminiscence protocol (Pierce). To detect PKR-associated viral nucleic acids, the PKR immunoprecipitate was subjected to proteinase K digestion and phenol extraction. Purified RNA was subjected to dot blot analysis with probes specific for HA vRNA as described below.

### Density gradient centrifugation

2.5×10^6^ A549 cells were mock-treated or infected with B/Lee WT or NS1 58/60/64 (#4) mutant virus at an MOI of 1 at 33°C. Cells were lyzed 12 hours p.i. in LyP-100 buffer (25 mM Hepes pH 7.5, 100 mM NaCl, 2.5 mM MgCl_2_, 0.1% Igepal-CA 630, 10 mM β-glycerophosphate, 2 mM Na_3_VO_4_, 1 mM Pefabloc, 5 µl/ml RNasin). Lysates were loaded onto a continuous sucrose gradient (5 to 50% in LyP-100 buffer) and centrifuged in a SW60Ti rotor (Beckman) for 150 minutes at 30.000 rpm and 4°C. Sixteen 250 µl fractions were taken from top to bottom. Fractions 1 to 9 were halved and analyzed by immunoblotting or subjected to RNA extraction (RNeasy, Qiagen), respectively. Denatured extracted RNA in 7.5% formaldehyde and 10xSSC were dotted onto Nylon transfer membrane and NS and HA vRNAs were detected with segment-specific DIG-labeled probes. DIG-labelled probes were generated from linearized template DNA by T7 RNA polymerase-mediated transcription using the DIG Northern Starter Kit (Roche).

### Leptomycin B (LMB) treatment of infected cells

1×10^6^ A549 cells were either mock-treated of infected with B/Lee WT or NS1 58/60/64 (#4) mutant virus at an MOI of 1. 7.5 ng/ml LMB (Sigma) or an equal volume of 70% methanol as a solvent control was added to the supernatant at 3 hours p.i.. At 8, 12 and 16 hours p.i. cells were lyzed in kinase binding buffer containing 0.5% Igepal-CA 630 and were analyzed by immunoblotting, as described above. The fractionation of cell lysate is described in the supporting materials and methods section (Text S1). In parallel, A549 cells grown on glass coverslips were infected and treated with LMB as described above. The cells were fixed with 2.5% paraformaldehyde and permeabilized with 0.2% Triton X-100 at 8, 12 or 16 hours. Viral NP was stained with primary mouse anti-NP antibody (AbD Serotec) and secondary goat anti-mouse Alexa 594 antibody (Molecular Probes). Cells were not stained for PKR as the available antibody appeared to be not suitable. Cells were analyzed with a LSM510 Meta confocal laser scanning microscope (Zeiss, Jena, Germany) equipped with a 63x/1.2 water objective lens. Data were analyzed and processed by the LSM Image Browser 3.5 and Adobe Photoshop 4.0 software packages.

### PKR autophosphorylation assay

5×10^6^ 293T cells were transfected with pcDNA3.1-PKR-V5/His or pcDNA3.1-PKR-K60A-V5/His, respectively. Cells were lyzed in kinase binding buffer containing 0.5% Igepal-CA 630, the cleared lysate was incubated with mouse anti-V5 antibody (AbD Serotec) and immunocomplexes were collected on protein G-agarose. The beads were washed twice with kinase binding buffer and twice with kinase assay buffer (20 mM Hepes pH 7.5, 50 mM KCl, 2 mM Mg(OAc)_2_, 2 mM MnCl_2_, 25 mM Na-β-glycerophosphat). The activation assay was performed on immobilized PKR in kinase assay buffer with 250 µM ATP, 10 µCi [γ-^32^P]-ATP, 5 mM Na_3_VO_4_, 1 mM NaF, 1 mM DTT. Viral RNPs were purified from detergent-treated virions as described in the supporting methods section (Text S1). PKR was activated by addition of pooled influenza B virus vRNP fraction, 2.5 ng/µl poly I∶C (Sigma) or 12.5 ng/µl heparin (Sigma), respectively, and incubated for 30 min at 30°C. Autophosphorylated PKR was analyzed by 10% SDS-PAGE and visualized by autoradiography. Densitometric evaluation was done with the AIDA 4.18.028 software and is given as fold activation compared to mock-treated PKR. For RNase treatment, vRNPs were incubated for 15 min at 37°C alone or with 5 U RNase III (Ambion) or 5 U RNase I (Ambion), respectively, prior to the kinase reaction. For PKR activation by synthetic vRNA, the plasmid pV-WT [57] was digested with *MboII* (Fermentas), treated with Klenow fragment (Fermentas) for 10 min at 37°C and used as a template for T7 RNA polymerase mediated *in vitro* transcription. The 53 nt long model vRNA containing the terminal 5′ and 3′ end sequences of influenza virus NS segment connected by a short linker was purified by a Quick Spin Oligo Column (Roche) and mock-treated or treated with Antarctic phosphatase (NEB) for 30 min at 37°C. The phosphatase was inactivated at 65°C for 15 min. vRNAs were purified by phenol/chloroform extraction and ethanol precipitation and concentrations were determined by UV spectroscopy. Precipitated PKR was incubated with mock-treated or phosphatase-treated vRNA for 30 min at 30°C. Autophosphorylation of PKR and densitometric analyses were done as described above.

## Supporting Information

Figure S1 Pathogenicity of influenza B viruses expressing dsRNA-binding defective NS1 proteins is enhanced in *PKR null* mice. (A) Groups of eight-week-old female PKR^−/−^ and wild type C57B6 mice were anesthetized and infected intranasally with 1×10^5^ pfu of the indicated recombinant influenza B/Lee virus. For monitoring of viral disease, 8 animals were weighed daily for two weeks, and mean percentage weight loss of each group was compared with the weight immediately prior to infection. Mice were euthanized when observed in extremis. (B) Survival rate of PKR^−/−^ mice after infection with recombinant influenza B viruses. Dead animals were scored daily and represented as the percentage of surviving animals.
(0.18 MB TIF)

Figure S2 Preparation of vRNPs from recombinant influenza B/Lee virus. Virus was grown in embryonated chicken eggs and subjected to lysis and centrifugation over a discontinuous glycerol gradient as described in Text S1. Fractions were taken from top to bottom and analyzed by SDS-PAGE and Coomassie blue staining (upper panel) and immunblotting with NP specific antibody (middle panel). RNA was extracted from the fractions and subjected to reverse transcription (RT)-PCR with primers specific for the vRNA of the NS segment (lower panel).
(0.58 MB TIF)

Figure S3 Treatment of infected cells with LMB inhibits the nuclear export of NP. A549 cells were infected with influenza B/Lee WT virus at an MOI of 1. Cells were mock treated or complemented with LMB starting at 3 hrs p.i.. Cells were lyzed at 15 hrs p.i. and cytoplasmic (C.) and nuclear fractions (N.) were generated. The fractions were analyzed by immunoblotting for viral NP and the marker antigens for the nuclear and cytoplasmic fractions, PARP and tubulin, respectively.
(0.11 MB TIF)

Text S1 This file contains supporting materials and methods.
(0.03 MB DOC)
