# Supplementary figures and images for: Influenza B Virus Ribonucleoprotein Is a Potent Activator of the Antiviral Kinase PKR
Source: PLoS Pathog. 2009 Jun 12;5(6):e1000473. doi: 10.1371/journal.ppat.1000473 (PMC2688073; doi:10.1371/journal.ppat.1000473)

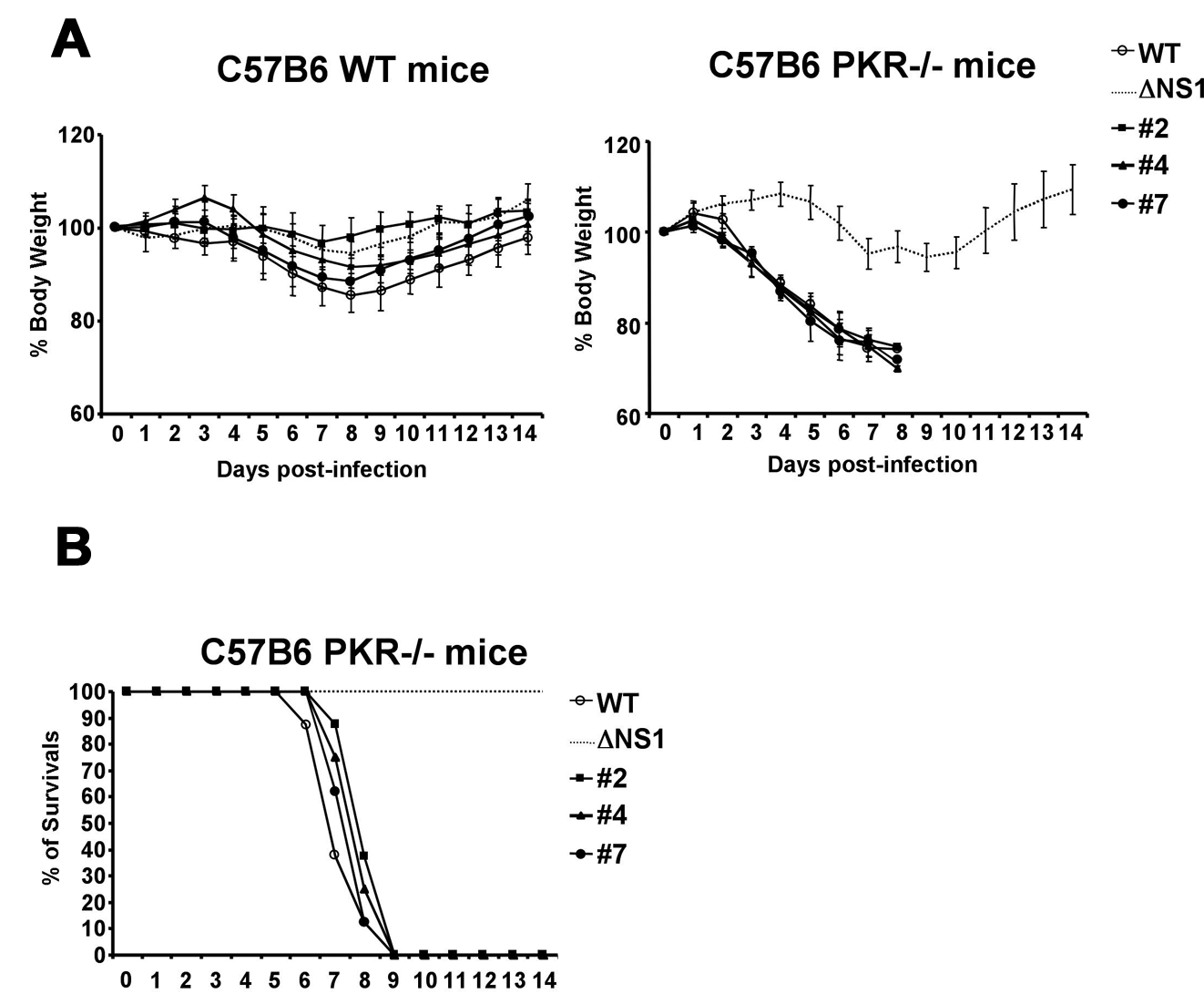

Supplement: Figure S1 — Pathogenicity of influenza B viruses expressing dsRNA-binding defective NS1 proteins is enhanced in PKR null mice. (A) Groups of eight-week-old female PKR−/− and wild type C57B6 mice were anesthetized and infected intranasally with 1×10^5^ pfu of the indicated recombinant influenza B/Lee virus. For monitoring of viral disease, 8 animals were weighed daily for two weeks, and mean percentage weight loss of each group was compared with the weight immediately prior to infection. Mice were euthanized when observed in extremis. (B) Survival rate of PKR−/− mice after infection with recombinant influenza B viruses. Dead animals were scored daily and represented as the percentage of surviving animals. (0.18 MB TIF) [file ppat.1000473.s001.tif]

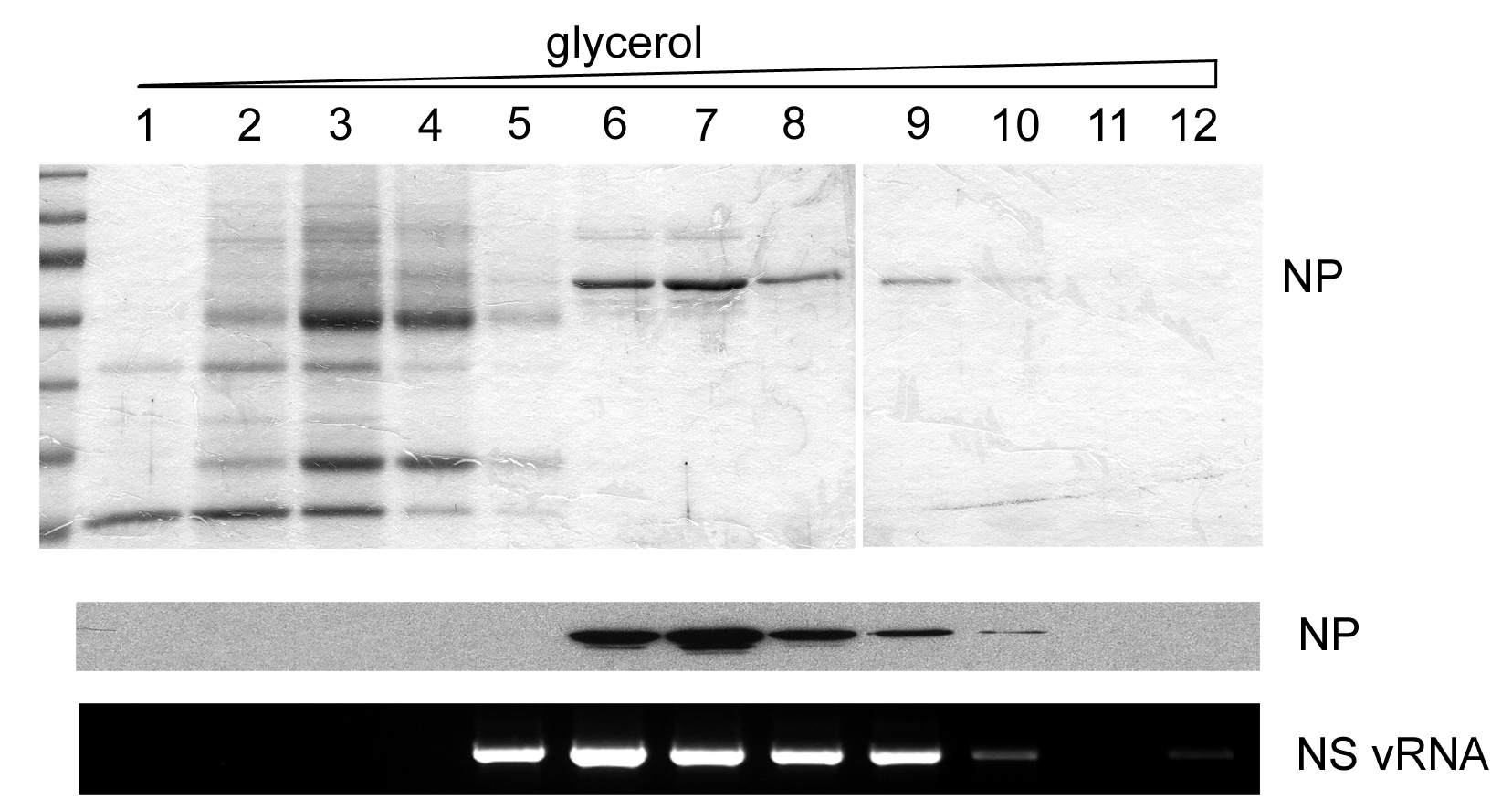

Supplement: Figure S2 — Preparation of vRNPs from recombinant influenza B/Lee virus. Virus was grown in embryonated chicken eggs and subjected to lysis and centrifugation over a discontinuous glycerol gradient as described in Text S1. Fractions were taken from top to bottom and analyzed by SDS-PAGE and Coomassie blue staining (upper panel) and immunblotting with NP specific antibody (middle panel). RNA was extracted from the fractions and subjected to reverse transcription (RT)-PCR with primers specific for the vRNA of the NS segment (lower panel). (0.58 MB TIF) [file ppat.1000473.s002.tif]

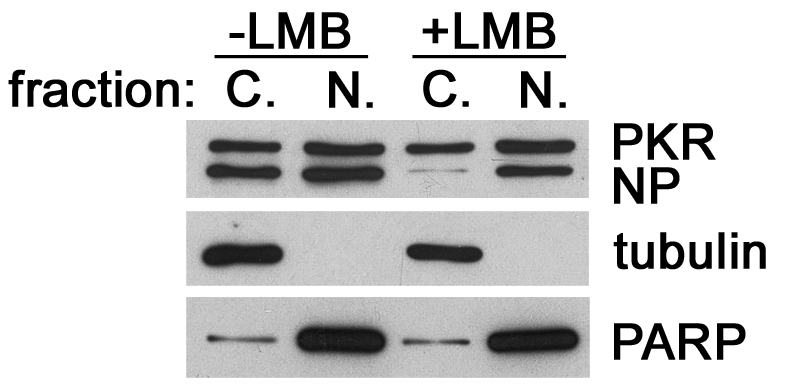

Supplement: Figure S3 — Treatment of infected cells with LMB inhibits the nuclear export of NP. A549 cells were infected with influenza B/Lee WT virus at an MOI of 1. Cells were mock treated or complemented with LMB starting at 3 hrs p.i.. Cells were lyzed at 15 hrs p.i. and cytoplasmic (C.) and nuclear fractions (N.) were generated. The fractions were analyzed by immunoblotting for viral NP and the marker antigens for the nuclear and cytoplasmic fractions, PARP and tubulin, respectively. (0.11 MB TIF) [file ppat.1000473.s003.tif]
