# Supplementary material for: Influenza B Virus Ribonucleoprotein Is a Potent Activator of the Antiviral Kinase PKR
Source: PLoS Pathog. 2009 Jun 12;5(6):e1000473. doi: 10.1371/journal.ppat.1000473 (PMC2688073; doi:10.1371/journal.ppat.1000473)
Supplement: Text S1 — This file contains supporting materials and methods. (0.03 MB DOC) [file ppat.1000473.s004.doc]

**SUPPORTING MATERIALS AND METHODS**

**Purification of vRNPs**

Recombinant influenza B/Lee wild-type virus was grown in the allantoic cavities of 11-day-old embryonated chicken eggs for 3 days at 33°C. The virus was purified and further concentrated by sedimentation through a 30% sucrose cushion during centrifugation in a SW32Ti rotor (Beckman) for 90 min at 26,000 rpm and 4°C. Virion RNPs were purified essentially as described elsewhere (Gomez-Puertas et al., 2000). In brief, the virus pellet was resuspended in 800µl disruption buffer (100mM Tris-HCl pH7.5, 100mM NaCl, 5mM MgCl_2_, 2% Triton X-100, 5% glycerol, 10mg/ml lysolecithin, 1.5 mM DTT) and incubated for 30 min at 30°C. The virus lysate was loaded onto a discontinuous glycerol gradient containing four steps of 800 µl of 70, 50, 40 and 33% glycerol in 50mM Tris-HCl pH7.5, 150 mMNaCl and centrifuged in a SW61Ti rotor (Beckman) for 245 min at 47,000 rpm and 4°C. Fractions of the gradient were collected from the top (fraction 1 to 12) and analyzed by SDS gel electrophoresis and Coomassie blue staining. RNA was extracted with the MinElute Virus Spin kit (Qiagen) and analyzed by RT-PCR with NS vRNA specific primers (Dauber et al., 2004). The vRNP containing fractions 6 and 7 were pooled and aliquots were stored at -80°C.

**Fractionation of cells after infection with influenza B virus in the presence or absence of LMB**

A549 cells grown in culture dishes were infected with WT virus at an MOI of 1. Cells were mock treated or complemented with 7.5 ng/ml LMB starting at 3 hrs p.i. At 15 h p.i. cells were swollen in hypotonic buffer A (10 mM Hepes, pH 8.0, 10 mM KCl, 0.1 mM EDTA, 0.1 mM EGTA, 1 mM Pefabloc, 1 mM Na_3_VO_4_) for 15 minutes on ice. Afterwards, cells were passaged ten times through a syringe attached to a 26Gx1’’ needle and centrifuged for 5 minutes at 5000 rpm. The supernatants were spun again at 14.000 rpm for 10 minutes to generate the cytosolic fraction. The pellet of the low speed centrifugation was washed twice with buffer A, resuspended in buffer B (20 mM Hepes, pH 8, 400 mM NaCl, 1 mM EDTA, 1 mM EGTA, 1 mM Pefabloc, 1 mM Na_3_VO_4_) and was incubated at 4° for 15 minutes. The suspension was centrifuged at 14.000 rpm for 10 minutes to generate the nuclear extract fraction. The fractions were analyzed by immunoblotting for viral NP and the marker antigens for nuclear and cytoplasmic fractions, PARP and tubulin, respectively.

**Cited literature**

Dauber, B., Heins, G., and Wolff, T. (2004). The influenza B virus nonstructural NS1 protein is essential for efficient viral growth and antagonizes beta interferon induction. J Virol *78*, 1865-1872.

Gomez-Puertas, P., Leahy, M.B., Nuttall, P.A., and Portela, A. (2000). Rescue of synthetic RNAs into thogoto and influenza A virus particles using core proteins purified from Thogoto virus. Virus Res *67*, 41-48.
